# *Helicobacter pylori* HP0231 Influences Bacterial Virulence and Is Essential for Gastric Colonization

**DOI:** 10.1371/journal.pone.0154643

**Published:** 2016-05-03

**Authors:** Yu Zhong, Florian Anderl, Tobias Kruse, Franziska Schindele, Elżbieta Katarzyna Jagusztyn-Krynicka, Wolfgang Fischer, Markus Gerhard, Raquel Mejías-Luque

**Affiliations:** 1 Institut für Medizinische Mikrobiologie, Immunologie und Hygiene, Technische Universität München, Munich, Germany; 2 Max von Pettenkofer-Institute for Hygiene and Medical Microbiology, Ludwig-Maximilians-Universität, Munich, Germany; 3 German Centre for Infection Research (DZIF), partner site Munich, Munich, Germany; 4 Department of Bacterial Genetics, Institute of Microbiology, Faculty of Biology, University of Warsaw, Warsaw, Poland; Institut Pasteur Paris, FRANCE

## Abstract

The Dsb protein family is responsible for introducing disulfide bonds into nascent proteins in prokaryotes, stabilizing the structure of many proteins. *Helicobacter pylori* HP0231 is a Dsb-like protein, shown to catalyze disulfide bond formation and to participate in redox homeostasis. Notably, many *H*. *pylori* virulence factors are stabilized by the formation of disulfide bonds. By employing *H*. *pylori* HP0231 deficient strains we analyzed the effect of lack of this bacterial protein on the functionality of virulence factors containing putative disulfide bonds. The lack of *H*. *pylori* HP0231 impaired CagA translocation into gastric epithelial cells and reduced VacA-induced cellular vacuolation. Moreover, *H*. *pylori* HP0231 deficient bacteria were not able to colonize the gastric mucosa of mice, probably due to compromised motility. Together, our data demonstrate an essential function for *H*. *pylori* HP0231 in gastric colonization and proper function of bacterial virulence factors related to gastric pathology.

## Introduction

*Helicobacter pylori* infection is one of the most common bacterial infections worldwide. Chronic gastritis induced by *H*. *pylori* represents the first step of a pathological cascade that can eventually progress to cancer [1,2]. In order to cope with the acidic conditions *H*. *pylori* encounters in the stomach, the bacterium produces urease, thereby hydrolyzing urea and increasing the pH locally [3]. In addition, motility mediated by flagella and cell morphology is essential for successful colonization of the gastric epithelium [4].

The immune response towards the bacterium as well as the degree of gastric pathology have been related to the presence of certain bacterial virulence factors. Of those, the Cag Pathogenicity Island (CagPAI) and VacA are considered the major pathogenic factors, and their presence defines populations at higher risk for gastric cancer development [5]. The CagPAI encodes CagA and several genes composing a type IV secretion system (T4SS), capable of translocating CagA into host epithelial cells. Once translocated, CagA interacts with a number of host proteins and activates signaling cascades that contribute to pathology [6]. Conversely, VacA is a secreted cytotoxin which is internalized, forming vacuoles in the host cells, and causing changes in mitochondrial potential [7] or disrupting autophagy [8].

Disulfide bonds, which in prokaryotes are introduced by proteins of the Dsb family, stabilize the structure of many proteins. Dsb proteins are crucial for correct folding or assembly of different pathogenic factors such as toxins, adhesins or components of type III secretion systems. Hence, loss of disulfide bond formation due to mutations in *dsb* genes were found to reduce pathogenesis of bacteria and viruses [9–12]. Dsb proteins have been largely studied in bacteria such as *E*. *coli*. In contrast, the disulfide bond formation machinery of *H*. *pylori* is still less characterized. *H*. *pylori* possesses different putative Dsb homologs: HP0595 (DsbI) was identified as a DsbB homolog, HP0377 as DsbC, and HP0265 as CcdA homolog [13,14]. However, it has been recently shown that HP0377 is a cytochrome c maturation (CcmG) protein playing also a role in Dsb isomerization pathway [15]. Initial structural studies proposed HP0231 as a DsbG homolog [16], however, more recent data showed HP0231 to have DsbA-activity [17,18]. Notably, disulfide formation mediated by HP0231 is important for folding, solubilization, production and secretion of *H*. *pylori* cysteine rich protein (HcpE), which is suggested to contribute to bacterial virulence [18]. Interestingly, presence of disulfide bonds has been reported in bacterial virulence factors such as VacA [19] or components of the T4SS [20], suggesting these are potential targets of the *H*. *pylori* Dsb system and specifically of HP0231.

In order to elucidate whether HP0231 is required for proper functionality of virulence factors containing disulfide bonds such as VacA and the T4SS, we generated HP0231-deficient *H*. *pylori* strains. In addition, we studied the impact of loss of HP0231 in gastric colonization in mice. Our results suggest an important role for HP0231 in the establishment of the infection and in determining bacterial virulence.

## Material and Methods

### Bacterial strains and growth conditions

*H*. *pylori* wild type P12 [21], G27 [22], PMSS1 and SS1 [23] or mutant strains PMSS1Δ*cagA*, PMSS1Δ*cagE* [24], P12Δ*vacA* [25], P12*ΔggT* [26], G27Δ*cagA* (generated by natural transformation of J99 Δ*cagA* DNA into *H*. *pylori* strain G27) and G27Δ*cagE* [27] were cultured on WC dent agar plates under microaerophilic conditions. All the bacterial strains used for experiments were sub-cultured no more than 3 times to avoid possible genotypic and phenotypic changes.

### Generation of *H*. *pylori* strains by natural transformation

Plasmids to generate HP0231 knockout strains and the corresponding HP0231 complemented strains were previously described [17]. To knock out *hp0231*, a PCR fragment, which was composed with the upstream region of *hp0231* gene followed by a chloramphenicol cassette and the downstream region of *hp0231* gene, was cloned into pGEM-T (Promega, USA) easy vector to generate the knockout plasmid pGMTeasy-Δ231. To complement the knockout strains with HP0231, the *hp0231* coding region was amplified from *H*. *pylori* 26695 genome DNA and was inserted into the shuttle vector pHeL3 to construct the vector pHeL3-231+. The plasmid was then introduced into knockout strains by natural transformation.

To naturally transform *H*. *pylori*, 10μg of plasmid DNA were introduced into around 4x10^8^ *H*. *pylori* bacterial cells during exponential growth phase. After one-day incubation on WC dent plates, bacteria were transferred onto WC dent plates containing antibiotics for selection. Single colonies were picked, grown and analyzed by PCR and western blot (S1A and S1B Fig). No changes in growth were detected between wild type and HP0231 knockout strains (S1C Fig).

### *H*. *pylori* growth curve

*H*. *pylori* cells growing at exponential phase on WC dent plates were resuspended in PBS and used to inoculate Brucella Broth dent liquid medium containing 10% FCS at an optical density (OD) of 0.5. Cultures were kept shaking under microaerophilic conditions. Samples were taken at different time points and cell density was determined by measuring OD_600_.

### Cell culture and infections

AGS (ATCC CRL-1739) and MKN45 [28] were cultured in DMEM (Gibco, USA) containing 10% FCS, at 37°C and 5%CO_2_. Cells were routinely tested for Mycoplasma contamination.

For infection experiments, 1x10^5^ cells were seeded on a well of a 24-well plate. After 24h, cells were infected with different *H*. *pylori* strains at MOI 20. After 6h infection, cells were photographed, supernatants collected and cell lysates obtained for western blot.

### Western blot

Cells were lysed using SDS sample buffer (62.5 mM Tris-HCl (pH 6.8), 2% w/v SDS, 10% glycerol, 50 mM DTT, 0.01% w/v bromophenol blue), while proteins from supernatants of OD 1 bacterial cultures were extracted after 24 hours incubation by TCA precipitation and resuspended in Laemmli buffer and 5xSDS-buffer as previously reported [29]. Equal amounts of lysate were loaded on SDS-PAGE gels, separated proteins were transferred to nitrocellulose membrane (Protran, Germany) and membranes blocked before applying primary antibodies. CagA phosphorylation was detected using a rabbit anti-CagA antiserum (kindly provided by Dr. R. Vogelmann, University Hospital Mannheim II, Mannheim, Germany), and a p-Tyr antibody (Millipore, USA). VacA antibody AK197, CagI antibody AK293 and CagL antibody AK271 have been previously described [30,31]. HP0231 was detected by using an anti-HP0231 serum (1:500) obtained after immunization of C57BL/6 mice with HP0231 recombinantly produced in our lab. β-actin was detected with anti-β-actin antibody (Sigma-Aldrich, USA).

### CagA translocation (β-lactamase activity)

TEM-1 β-lactamase activity was determined as described elsewhere [32]. Briefly, AGS cells were infected with *H*. *pylori* P12 [TEM-1-CagA] for 2.5 hours, and cells were loaded subsequently with the fluorescent substrate CCF4-AM in the respective loading solution (LiveBLAzer-FRET B/G loading kit; Invitrogen) supplemented with 1 mM probenecid (Sigma). Cells were incubated at room temperature in the dark for 90 min. For quantification of translocation, infected cells were measured with a Clariostar plate reader (BMG Labtech) using an excitation wavelength of 405 nm (10 nm bandwidth), and emission at 460 nm (20 nm bandwidth, blue fluorescence) and 530 nm (15 nm bandwidth, green fluorescence). CagA translocation was defined as the emission ratio at 460 nm_(sample-blank)_ divided by 530 nm_(sample-blank)_ (Blue-to-Green ratio).

### ELISA

1x10^5^ AGS cells were infected by different *H*. *pylori* strains and the supernatant was collected after 6h infection. The secretion of Interleukin-8 (IL8) was detected using IL-8 Elisa kit, according to manufacturer’s instructions (eBioscience, USA).

### Vacuolation Assay

Cell vacuolation was monitored by counting vacuole formation in *H*. *pylori-*infected AGS cells. Briefly, 5x10^4^ AGS cells were seeded into 6-well plates and infected with different *H*. *pylori* strains at MOI 100 for 24h. High magnification pictures (400x) were taken at five random positions for each sample. Cells that showed vacuoles were counted and percentage of cells showing vacuoles in total cell number was calculated.

### *H*. *pylori* gamma-glutamyltranspeptidase activity assay

*H*. *pylori* gamma-glutamyltranspeptidase activity was monitored by following the cleavage of g-glutamyl-p-nitroanilide (gGpNA). 10^8^ cells of different *H*. *pylori* strains were resuspended in 200 μl of Tris-buffer pH8.0, which contained 20 mmol/L glycyl-glycine, 2.5 mmol/L L-γ-glutamyl-p-nitroanilide (Sigma, USA) and incubated at 37°C for 10min. The release of p-nitroanilide was then detected by spectrophotometry at 405nm. All assays were performed in triplicates.

### Bacterial Binding Assay

Human gastric cancer cells were seeded in 96-well plates (1.5x10^5^ cells per well). Different *H*. *pylori* strains were labeled by cell tracer CFDA-SE (Life Technologies, USA) in PBS at 37°C for 30min. After washing three times with PBS, labeled *H*. *pylori* were added at MOI 10 to the cells and incubated for 1h at 37°C. Samples were washed with PBS three times and fixed with 100μL 4% paraformaldehyde (PFA) for 5min. Finally, the samples were resuspended with 200μL FACS buffer and analyzed by flow cytometry. Analysis was performed with a FACS CyAn (Beckman Coulter) and the FlowJo software.

### Immunofluorescence

5x10^4^ AGS cells were seeded on a well of 4-well chamber slide (Thermo Fisher Scientific, USA). After 24h, AGS cells were infected by CFDA-SE labeled *H*. *pylori* PMSS1 or PMSS1Δ*hp*0231 at MOI 5 for 1h at 37°C. Samples were washed 5 times with PBS and fixed with 4% PFA for 10min. After washing twice with PBS, samples were stained with pre-warmed deep red/PBS (1:1000 dilution; Thermo Fisher Scientific, USA) for 30min at 37°C. After washing with PBS, samples were mounted with DAPI (Vector laboratories, USA) and fluorescence pictures were taken by confocal microscopy (Olympus Life Science, Japan) (600x magnification).

### Motility Assays

*H*. *pylori* motility was assessed on soft agar plates as previously described [17,33] with slightly modifications. Briefly, the soft agar plates used in this study were composed of 0.35% (w/v) agar, 10% (v/v) FCS, 2.8%(w/v) Brucella Broth base, 5 μg/mL trimethoprim, 5 μg/mL amphotericin B, 10 μg/mL vancomycin and 5 μg/mL cefsulodin. *H*. *pylori* were inoculated with a sterile pipette tip into a soft agar plate. Migration of the bacteria on the plate was monitored and pictures were taken after 2 to 3 days of culture.

### Mouse experiments

6–8 weeks old C57BL/6 mice were used for *H*. *pylori* infection. Mice were orally infected with 4x10^8^ bacteria three times every two days and sacrificed after 7 days or 1 month. Stomach pieces were weighted and homogenized in Brain-Heart infusion medium. Gastric homogenate dilutions were plated on WC dent plates containing 200 μg/mL bacitracin, 10 μg/mL nalidixic acid and 3 μg/mL polymyxin B and antibiotics for selection. After 4 to 6 days incubation, *H*. *pylori* colonies were counted.

### Ethics statement

All animal studies were conducted in compliance with European guidelines for the care and use of laboratory animals and were approved by the Government of Oberbayern (AZ- 55.2-1-54-2532-147-12).

## Results

### HP0231 is essential for CagA translocation

**Fig 1:**
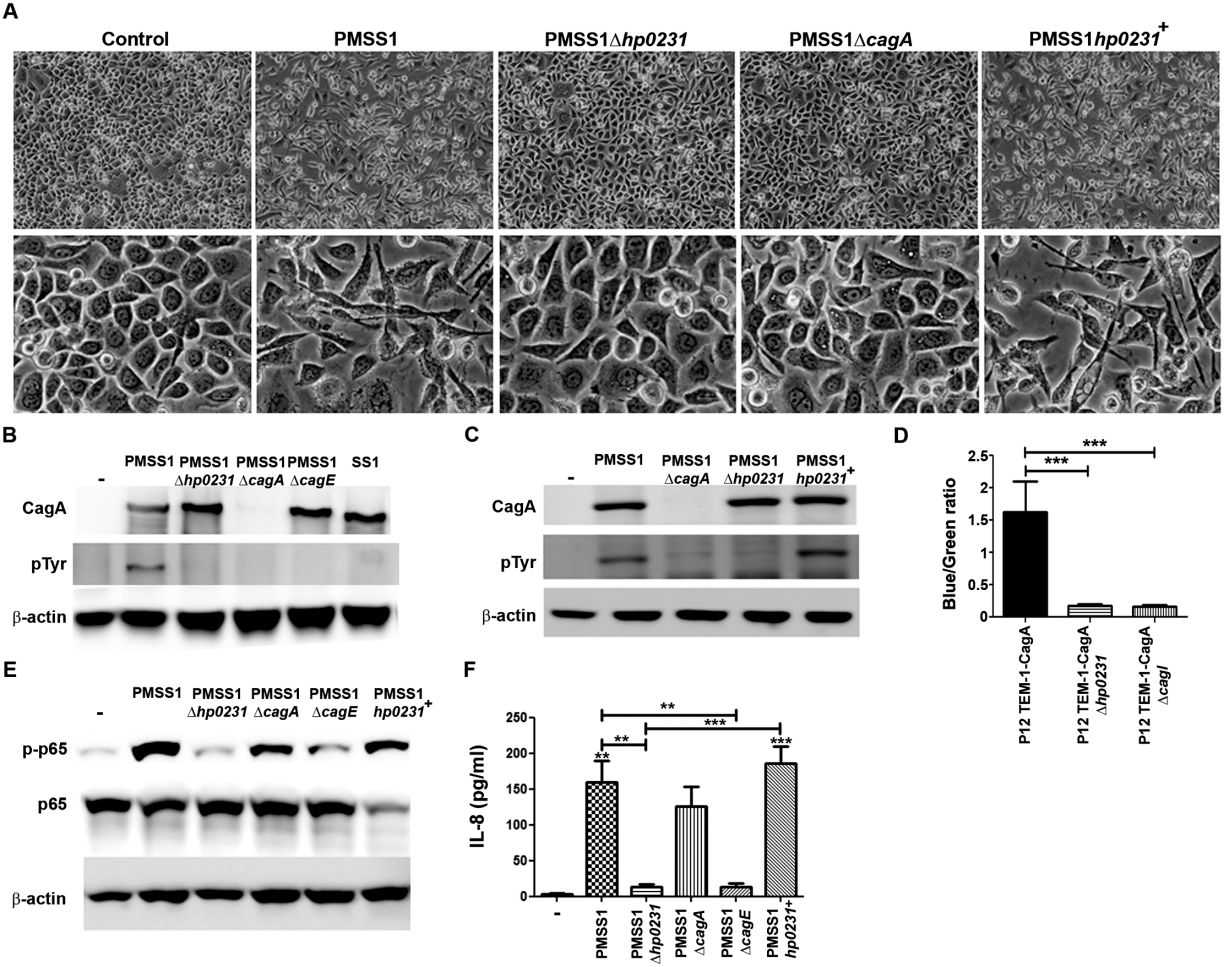
Lack of *H*. *pylori* HP0231 impairs CagA translocation. (A) Representative pictures of AGS cells infected with the *H*. *pylori* strain PMSS1 and the indicated isogenic mutant strains at MOI 20. Pictures of the cultures were taken after 6 hours of infection at 100x (upper panel) and 400x (lower panel) magnification. (B) and (C) CagA phosphorylation detected by western blot after infection of AGS cells with wild type *H*. *pylori* PMSS1 and isogenic mutant strains for 6 hours at MOI 20. β-actin was used as a protein loading control. One representative blot from three independent experiments is shown. (D) CagA translocation measured as β-lactamase activity in cells infected with *H*. *pylori* strains producing TEM-1-CagA. The numbers indicated are mean values ± S.D. of blue-to-green fluorescence ratios obtained from four independent experiments performed in duplicate. ***p≤0.001; ANOVA, Bonferroni’s multiple comparison test. (E) Levels of phosphorylated and total p65 detected by western blot in lysates from AGS cells infected for 6 hours with the indicated *H*. *pylori* strains at MOI 50. One representative blot from three independent experiments is shown. (F) IL-8 secretion after infection of AGS cells for 6 hours with the indicated *H*. *pylori* strains at MOI 50. Results of three independent experiments expressed as mean ± S.D. are shown. **p≤0.01, ***p≤0.001; ANOVA, Bonferroni’s multiple comparison test. Asterisks on top of the bars indicate significances relative to uninfected cells.

As proteins composing the *H*. *pylori* T4SS were suggested to form disulfide bonds to maintain structural integrity, we sought to investigate whether lack of HP0231 could alter T4SS functionality by analyzing CagA translocation into gastric epithelial cells. To this end, we deleted *hp0231* in different *H*. *pylori* strains (S1A and S1B Fig) and used them for infection of AGS cells. *H*. *pylori* wild type bacteria induced a hummingbird phenotype (Fig 1A and S2A Fig). No such phenotypic changes were induced in cells infected with bacteria lacking *hp0231* or when using an isogenic strain lacking CagA, whereas complementation of *hp0231* reconstituted cell elongation. These data suggested impaired CagA translocation in the absence of HP0231. To further analyze CagA translocation after *hp0231* deletion, lysates from infected cells were subjected to western blot to detect CagA phosphorylation. Infection of cells with wild type bacteria resulted in CagA tyrosine phosphorylation (Fig 1B and S2B Fig), while in the absence of *hp0231*, phosphorylation of CagA could not be detected. As expected, CagA was not phosphorylated after infecting the cells with strains lacking *cagA* or *cagE* or infected with *H*. *pylori* SS1 (Fig 1B).

Since genetic manipulation of *H*. *pylori* often leads to secondary genetic changes resulting in impairment of CagA translocation, cells were infected with bacteria in which the *hp0231* gene had been complemented. In contrast to *hp0231* deficient bacteria, *hp0231*-complemented *H*. *pylori* strains translocated CagA, as detected by CagA phosphorylation (Fig 1C and S2C Fig), excluding off-target effects after deletion of *hp0231*.

Defects on CagA translocation were further confirmed by performing CagA translocation assays using a β-lactamase activity reporter assay. β-lactamase activity was observed when using CagA from wild type *H*. *pylori*, while no activity was detected when CagA from the *hp0231* isogenic knockout strain was analyzed (Fig 1D and S2D Fig).

The presence of a functional T4SS has been related to the activation of different signaling cascades in gastric epithelial cells. Thus, we next analyzed whether lack of *hp0231* affected T4SS-mediated signaling and focused on canonical NF-κB. Infection of AGS cells with wild type bacteria induced phosphorylation of p65 (Fig 1E), indicating activation of NF-κB upon infection. Notably, p65 phosphorylation was not detected in cells infected with bacteria lacking *hp0231*. Similar results were obtained when infecting the cells with bacteria lacking *cagE*, as expected, while the absence of *cagA* still led to p65 phosphorylation (Fig 1E). Complementation of the gene in the knockout strain led to activation of NF-κB at similar levels as observed in the wild type situation (Fig 1E), confirming that absence of *hp0231* attenuates host signaling events induced by *H*. *pylori*.

As activation of NF-κB by *H*. *pylori* initiates a pro-inflammatory response, mainly translating into IL-8 expression by gastric epithelial cells, we assessed the effect of deleting *hp0231* in IL-8 secretion. Cells infected with wild type *H*. *pylori* PMSS1 and the *cagA* deficient isogenic strain secreted IL-8 in response to the bacteria (Fig 1F). Upon deletion of *hp0231* only slight levels of IL-8 were detected, similar to the levels observed after infection with bacteria lacking *cagE*. Complementation of *hp0231* again restored IL-8 secretion (Fig 1F).

The T4SS is a protein complex spanning both bacterial membranes and containing several essential protein components. We have shown previously that two of these proteins, CagI and CagL, are produced at strongly reduced levels when genes encoding several other secretion apparatus proteins are missing, suggesting that production of normal CagI and CagL levels depends on correct assembly of these other components within the secretion apparatus [31]. Interestingly, we found that CagI and CagL are strongly reduced in levels after deletion of *hp0231* (S2E and S2F Fig), indicating that *hp0231* is also involved in correct assembly of the secretion apparatus.

Together, these results show that lack of *hp0231* alters *H*. *pylori* T4SS functionality, thereby not only hampering CagA translocation into gastric epithelial cells but also affecting pro-inflammatory responses towards the bacterium.

### Absence of HP0231 reduces *H*. *pylori*-induced vacuolation in host cells

**Fig 2:**
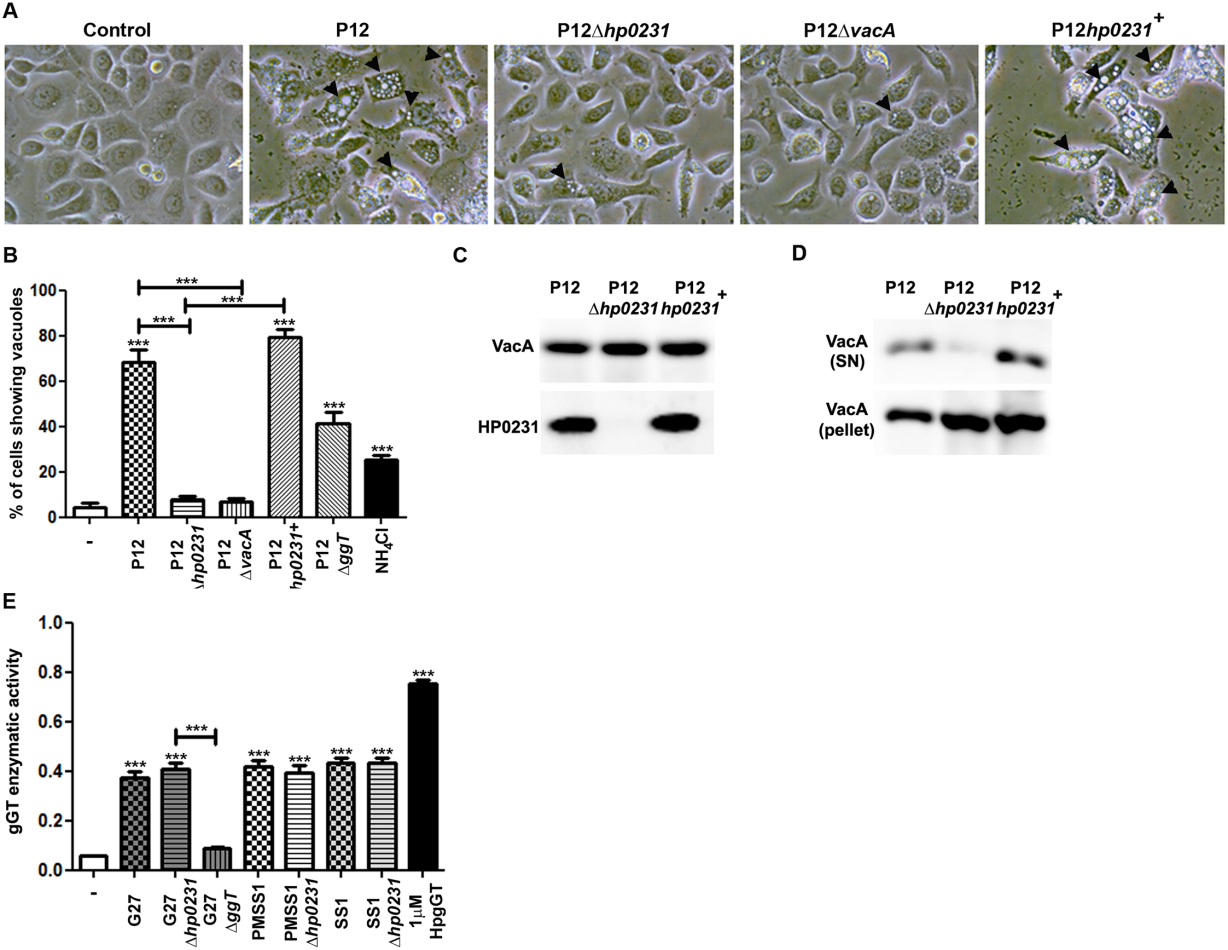
Lack of HP0231 reduces *H*. *pylori* vacuolation capacity. (A) Representative pictures of AGS cells infected with different H. pylori strains for 24 hours at MOI 100. Arrows indicate vacuoles. (400x magnification). (B) Percentage of cells showing vacuoles in response to *H*. *pylori* infection. NH_4_Cl was used as positive control. Five high power field (400x) were counted for each experiment. Results (mean ± S.D.) of three independent experiments are shown. ***p≤0.001; ANOVA, Bonferroni’s multiple comparison test. Asterisks on top of the bars indicate significances relative to uninfected cells. (C) VacA and HP0231 expression detected by western blot. (D) VacA levels in supernatants (SN) and pellets of 24 hours *H*. *pylori* cultures detected by western blot. (E) *H*. *pylori* gGT activity assay. G27Δ*ggt* was used as negative control, while recombinant gGT (HpgGT) was used as a positive control. Results from three independent experiments (mean ± S.D.) are shown. ***p≤0.001; ANOVA, Bonferroni’s multiple comparison test. Asterisks on top of the bars indicate significances relative to uninfected cells.

VacA is another major virulence determinant of *H*. *pylori* potentially forming disulfide bonds. Therefore, we analyzed whether lack of *hp0231* influenced VacA functionality *in vitro* by comparing cellular vacuolation upon infection with wild type and knockout bacteria. Infection with wild type bacteria resulted in vacuole formation in host cells (Fig 2A and 2B) that was reduced in the absence of *hp0231* to a similar extent as when infecting cells with *VacA* deficient bacteria. Vacuolation was restored when *hp0231* was complemented in the knockout strain (Fig 2A and 2B). Notably, the bacteria lacking *hp0231* expressed VacA at similar levels as wild type bacteria (Fig 2C). However, lack of *hp0231* highly impaired VacA secretion (Fig 2D), indicating that defects on HP0231 alter VacA secretion but not its expression.

To exclude unspecific effects of deletion of *hp0231*, we analyzed whether lack of *hp0231* also altered the function of other virulence factors. Specifically, we focused on gGT, since no disulfide bonds have been reported in its structure [34]. No changes in gGT activity were detected when *hp0231* was depleted (Fig 2E), confirming that the effects induced by HP0231 are specific for bacterial factors forming disulfide bonds.

### HP0231 is required for gastric colonization

**Fig 3:**
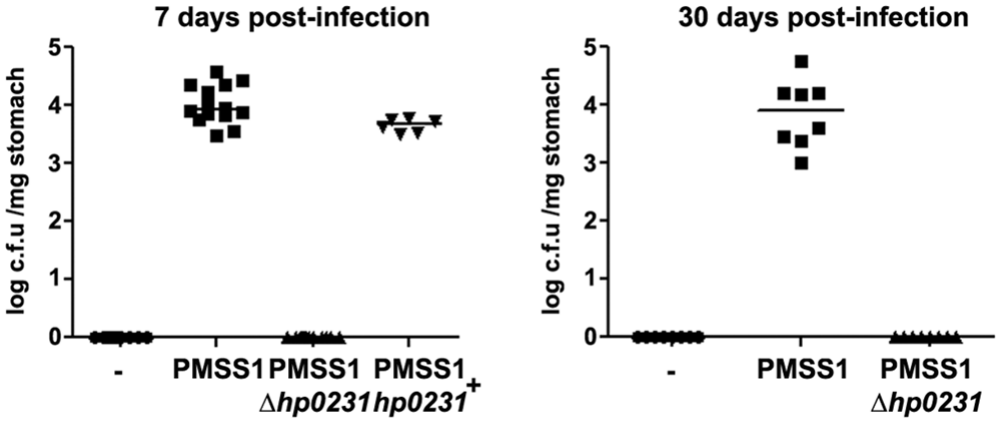
*H*. *pylori* HP0231deficient bacteria cannot colonize the gastric mucosa of mice. C57/BL6 mice were infected with *H*. *pylori* PMSS1 wild type or isogenic strains lacking or showing complementation of the *hp0231* gene. Colony forming units (c.f.u) were examined after plating serial dilutions of stomach homogenates. Each dot represents one mouse. Horizontal bars indicate medians.

Next, we examined the effects of deleting *hp0231* in an infection model *in vivo*. C57BL/6 mice were infected for 7 and 30 days and gastric colonization was assessed by counting colony formation from stomach homogenates. Bacteria lacking *hp0231* were not able to colonize the stomach of mice at either time point of analysis (Fig 3 and S3A Fig), while complementation of the gene reverted the phenotype observed. These results indicate that HP0231 is essential for gastric colonization.

### Lack of HP0231 does not affect bacterial binding but impairs motility

**Fig 4:**
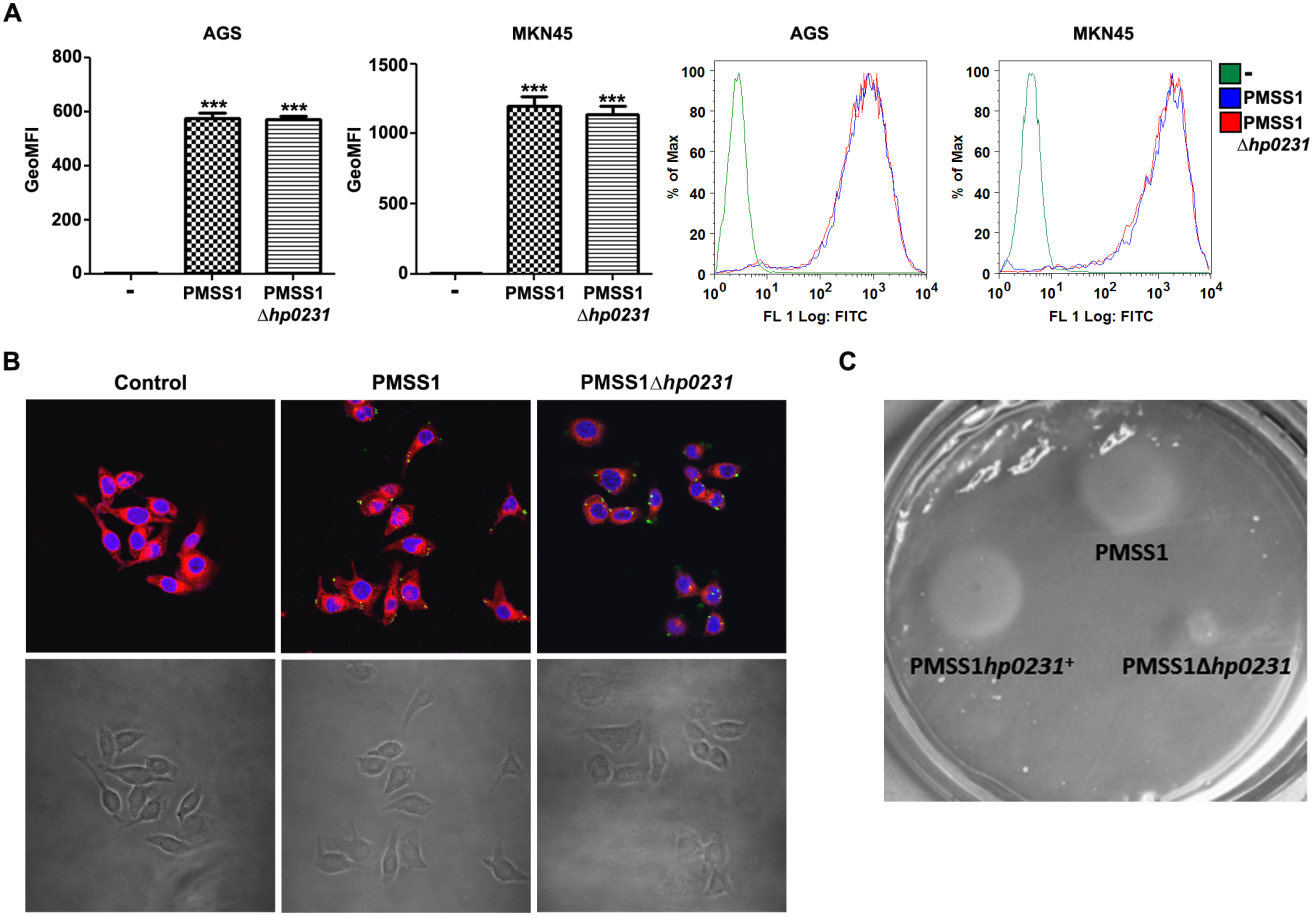
HP0231 does not alter bacterial binding but it alters motility. (A) Human gastric cancer epithelial cells were infected with CFDA-SE-labeled *H*. *pylori* PMSS1 or the PMSS1*hp0231* mutant strain and binding was analyzed by FACS. Geometric Mean Fluorescence Intensity (GeoMFI) from three independent experiments (left) and a representative histogram (right) are shown. ***p≤0.001; ANOVA, Bonferroni’s multiple comparison test. Asterisks on top of the bars indicate significances relative to uninfected cells. (B) Immunofluorescence and corresponding bright field pictures of AGS cells co-incubated for 1 hour with the indicated *H*. *pylori* strains (green). Nuclei were stained with DAPI (blue) and cell membranes with deep red (red). Magnification 600x. (C) *H*. *pylori* motility assessed after 2 days incubation on soft agar plates.

To colonize the gastric epithelium, *H*. *pylori* needs to bind to epithelial cells using different adhesins. As mice infected with *hp0231* deficient bacteria were not colonized, we first analyzed whether lack of *hp0231* induced defects in *H*. *pylori* binding to epithelial cells. Thus, human gastric epithelial cells were incubated with fluorescence-labeled bacteria and binding was determined by flow cytometry and immunofluorescence. Lack of *hp0231* did not affect binding of *H*. *pylori* to gastric epithelial cells, since no differences in binding were observed between wild type and *hp0231* knockout bacteria (Fig 4A and 4B), while lack of the adhesins BabA and SabA slightly reduced bacterial binding (S3B Fig).

As no differences in bacterial binding to host cells were detected, we then analyzed whether defects in motility could explain the lack of gastric colonization observed for the *hp0231* knockout strains. Indeed, in the absence of *hp0231 H*. *pylori* showed reduced motility, as previously reported in other bacterial backgrounds [17] (Fig 4C and S3C Fig), which may explain the defects in colonization detected.

## Discussion

Disulfide bond formation is an essential post-translational modification to maintain the proper conformational structure of many proteins. In bacteria, the Dsb system is mainly responsible for introducing disulfide bonds, however, not much data on Dsb proteins of *H*. *pylori* have been reported to date. Recently, *H*. *pylori* HP0231 was reported as a dimeric oxidoreductase that catalyzes disulfide bond formation in the periplasm [17]. In addition, it was found that HP0231 is important for folding and secretion of HcpE, a protein suggested to play a role in virulence [18]. As other virulence factors of *H*. *pylori* were suggested to form disulfide bonds, we analyzed whether HP0231 was important to maintain their functionality. Thus, we first focused on the T4SS. We observed that CagA translocation was impaired in the absence of *hp0231*, suggesting that lack of *hp0231* may destabilize the structure of the T4SS and therefore, virulence factors as CagA are prevented from being injected into host cells.

Two cysteine residues (C771 and C782) located near the C terminus of the p55 subunit of VacA were shown to form a disulfide bond [19], while mutation of either of these cysteines to serine resulted in decreased secretion of VacA [35]. Upon deletion of *hp0231*, we observed impaired vacuolating activity, which may be attributed to the loss of the disulfide bond stabilizing both cysteines important for the secretion of the toxin. Indeed, impaired VacA secretion was detected in bacteria lacking *hp0231*.

Our observations not only would strengthen the crucial role previously suggested for HP0231 in bacterial virulence, but also indicate that the functionality of two of the most important *H*. *pylori* virulence determinants is highly dependent on their correct folding controlled by HP0231. Nevertheless, structural studies would be necessary in order to identify specific conformational changes in bacterial proteins upon deletion of *hp0231*. Although *H*. *pylori* might possess other proteins involved in disulfide bond formation, our data indicate that they cannot compensate for the lack of *hp0231* concerning functionality of the T4SS and VacA.

Studies examining the role of another Dsb protein of *H*. *pylori* (DsbI) showed that impairment of disulfide bond formation highly reduced the ability of *H*. *pylori* to colonize the stomach of mice [36]. In line, we observed that bacteria lacking *hp0231* were not able to colonize the gastric mucosa of mice. Impaired colonization was not due to reduced binding of *H*. *pylori* to gastric epithelial cells, but most likely to compromised motility of the *hp0231* deficient bacteria. Defects in motility were already reported for the *H*. *pylori* strain N6 when *hp0231* was deleted [17]. Our results indicate that this is not a strain specific effect, but that HP0231 is important to maintain bacterial motility in general. Notably, the defects on motility observed for the *hp0231* deficient bacteria were not attributed to defects in the structure or distribution of the flagella but to changes in the morphology of the cells [17]. These morphological changes concomitant to a lack of motility would highly hamper colonization, and thus the bacteria would be rapidly cleared.

*H*. *pylori* infection is still a highly prevalent infection worldwide and current therapeutic strategies based on antibiotic treatment have failed mostly due to an increased rate of antibiotic resistance. Thus, the search of novel therapies based on specific inhibitors represents an interesting alternative to antibiotics. Our data suggest that HP0231 might potentially be a good target to develop inhibitors for the treatment of *H*. *pylori* infection. Blocking HP0231 in bacteria might induce morphological and structural changes, which, in the one hand, would reduce bacterial virulence once the bacterium has colonized the stomach and, on the other hand, would contribute to bacterial clearance.

Together, we provide evidence of a novel and important role for *H*. *pylori* HP0231 in bacterial virulence and colonization of the gastric mucosa. Moreover, our results should encourage the development of specific Dsb inhibitors to treat this widespread infection.

## Supporting Information

S1 Fig Characterization of *hp0231* Knockout and complemented bacteria. (A) Expression of *hp0231* assessed by PCR. bp, base pair. (B) Expression of HP0231 detected by western blot. CagA was used as a control. (C) *H*. *pylori* growth curve. Growth was determined by measuring optical density at the indicated times. Results from one representative experiment conducted in triplicate is shown.
(TIF)

S2 Fig Lack of HP0231 impairs CagA translocation. (A) Representative pictures of AGS cells infected with the indicated *H*. *pylori* strains for 6 hours at MOI 20. (B) and (C) CagA phosphorylation detected by western blot in AGS cells infected for 6 hours with *H*. *pylori* at MOI 20. β-actin was used as protein loading control. (D) TEM-1-CagA expression levels detected by western blot. (E) CagI and CagL protein expression levels detected by western blot. Arrows denote specific bands.
(TIF)

S3 Fig Lack of HP0231 impairs gastric colonization. (A) C57/BL6 mice were infected with *H*. *pylori* SS1 wild type or the isogenic strain lacking the *hp0231*. Colony forming units (c.f.u) were examined after plating serial dilutions of stomach homogenates. Each dot represents one mouse. Horizontal bars indicate medians. (B) Human gastric cancer epithelial cells were infected with CFDA-SE-labeled *H*. *pylori* G27 or isogenic mutant strains and binding was analyzed by FACS. Cells were gated on FSC/SSC followed by live/dead discrimination. Geometric Mean Fluorescence Intensity (GeoMFI) from three independent experiments are shown. **p≤0.01, ***p≤0.001; ANOVA, Bonferroni’s multiple comparison test. Asterisks on top of the bars indicate significances relative to uninfected cells. (C) *H*. *pylori* motility was assessed after 2 days incubation on soft agar plates.
(TIF)
