# Supplementary figures and images for: Helicobacter pylori HP0231 Influences Bacterial Virulence and Is Essential for Gastric Colonization
Source: PLoS One. 2016 May 3;11(5):e0154643. doi: 10.1371/journal.pone.0154643 (PMC4854439; doi:10.1371/journal.pone.0154643)

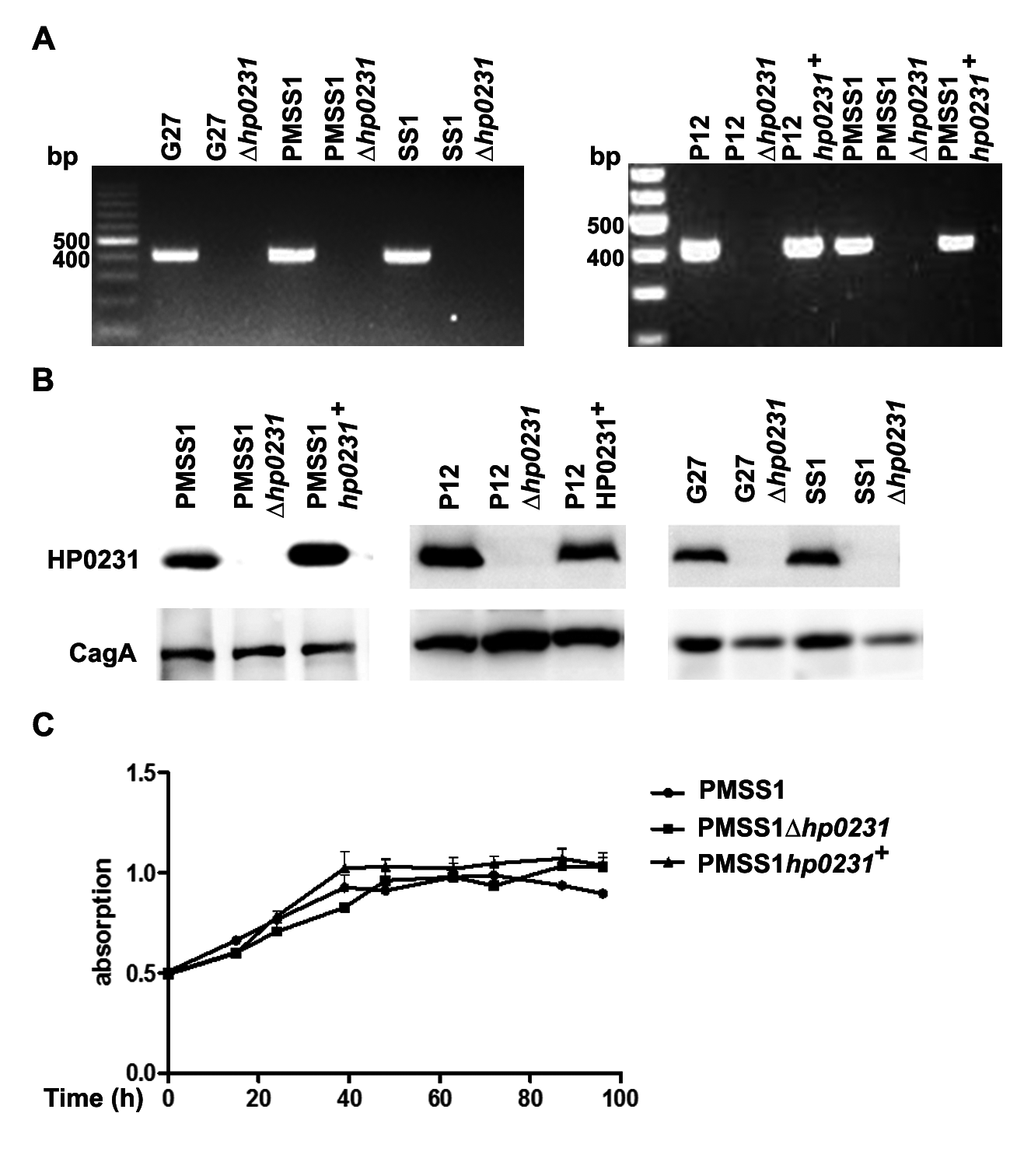

Supplement: S1 Fig — (A) Expression of hp0231 assessed by PCR. bp, base pair. (B) Expression of HP0231 detected by western blot. CagA was used as a control. (C) H. pylori growth curve. Growth was determined by measuring optical density at the indicated times. Results from one representative experiment conducted in triplicate is shown. (TIF) [file pone.0154643.s001.tif]

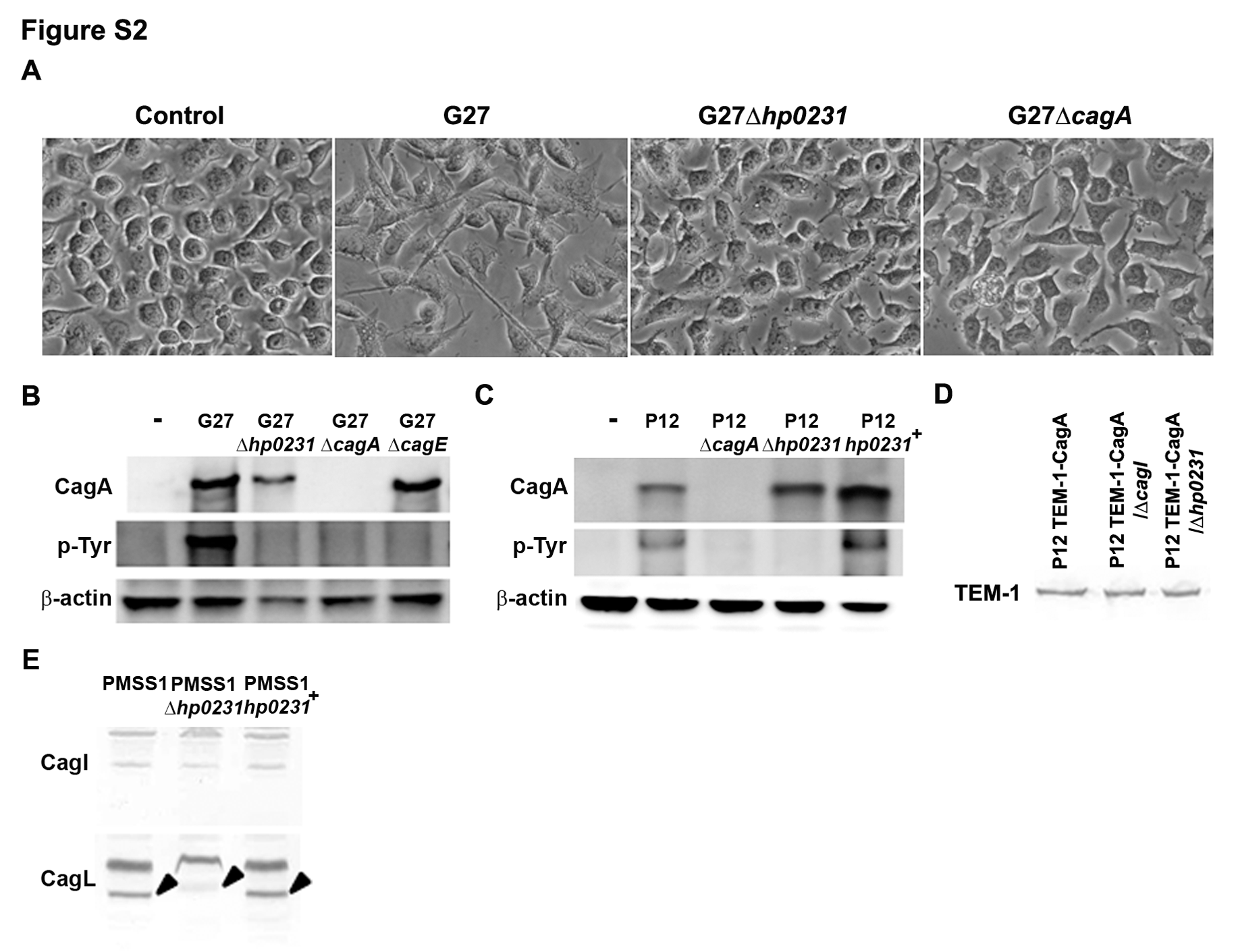

Supplement: S2 Fig — (A) Representative pictures of AGS cells infected with the indicated H. pylori strains for 6 hours at MOI 20. (B) and (C) CagA phosphorylation detected by western blot in AGS cells infected for 6 hours with H. pylori at MOI 20. β-actin was used as protein loading control. (D) TEM-1-CagA expression levels detected by western blot. (E) CagI and CagL protein expression levels detected by western blot. Arrows denote specific bands. (TIF) [file pone.0154643.s002.tif]

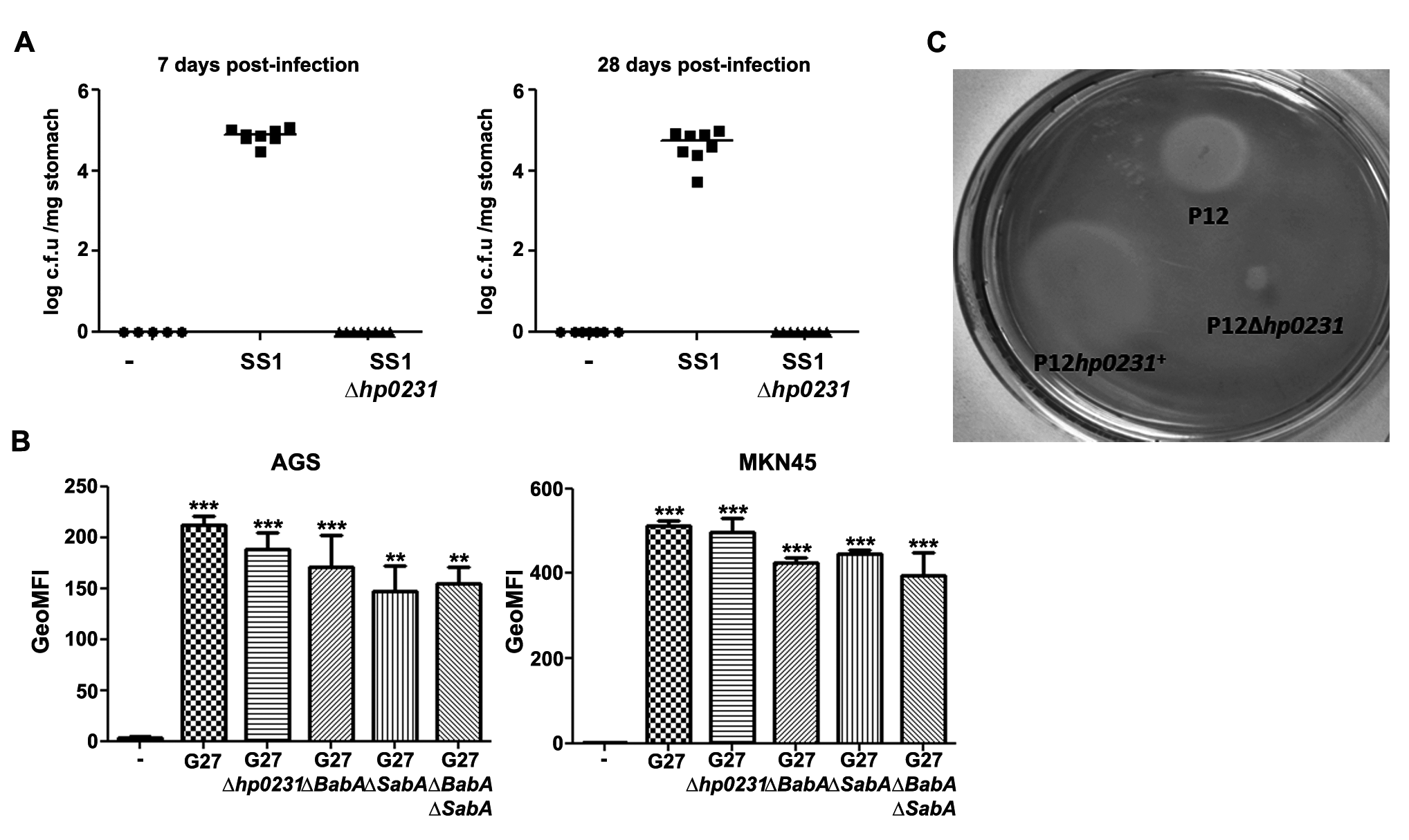

Supplement: S3 Fig — (A) C57/BL6 mice were infected with H. pylori SS1 wild type or the isogenic strain lacking the hp0231. Colony forming units (c.f.u) were examined after plating serial dilutions of stomach homogenates. Each dot represents one mouse. Horizontal bars indicate medians. (B) Human gastric cancer epithelial cells were infected with CFDA-SE-labeled H. pylori G27 or isogenic mutant strains and binding was analyzed by FACS. Cells were gated on FSC/SSC followed by live/dead discrimination. Geometric Mean Fluorescence Intensity (GeoMFI) from three independent experiments are shown. **p≤0.01, ***p≤0.001; ANOVA, Bonferroni’s multiple comparison test. Asterisks on top of the bars indicate significances relative to uninfected cells. (C) H. pylori motility was assessed after 2 days incubation on soft agar plates. (TIF) [file pone.0154643.s003.tif]
